# Thyroid hemiagenesis and Hashimoto’s thyroditis—diagnostic and treatment pitfalls

**DOI:** 10.1186/s12957-017-1250-0

**Published:** 2017-10-06

**Authors:** Minghao Wang, Lingmi Hou, Maoshan Chen, Lin Ren, Peng Tang, Yi Zhang, Jun Jiang

**Affiliations:** 1 Center of Breast Disease, Southwest Hospital, Third Military Medical University, Chongqing, 400038 China; 2 Department of Breast and Thyroid Surgery, Affiliated Hospital of North Sichuan Medical College, Nanchong, Sichuan 637000 China; 3 Department of Breast and Thyroid Surgery, Suining Central Hospital, Suining, Sichuan 629000 China

**Keywords:** Thyroid hemiagenesis, Hashimoto’s thyroiditis, Parathyroid gland

## Abstract

**Background:**

Thyroid hemiagenesis (TH) is a rare congenital disease with absence of a thyroid lobe; most patients have no clinical symptoms. The etiology of TH remains unclear. In this paper, we describe a rare case of TH and congenital absence of the ipsilateral parathyroid gland, found during the operation, combined with the autoimmune disease Hashimoto’s thyroiditis, also known as chronic lymphocytic thyroiditis.

**Case Presentation:**

A 31-year-old woman was admitted to our hospital because of a mass in the right neck. Surgical exploration validated the absence of the left lobe of the thyroid and parathyroid glands, and pathological examination of the excised nodules confirmed Hashimoto’s thyroiditis.

Patients with TH might show accompanying absence of the ipsilateral parathyroid gland. The case described here, in which TH was combined with Hashimoto’s thyroiditis, is rare in the medical literature. The operation should be ended at once if Hashimoto’s thyroiditis is diagnosed during surgery.

**Conclusions:**

Absence of thyroid lobe may accompany with a congenital absence of the ipsilateral parathyroid gland and Hashimoto’s thyroiditis. Fine needle aspiration is essential to diagnosis and decision-making of the treatment.

## Background

Thyroid hemiagenesis (TH), first reported by Handsfield-Jones in 1866, is a rare congenital abnormality with a low population incidence of about 0.05–0.2% [1–4]. To date, the cause of this congenital disease has not been clarified; it is usually the left lobe that is absent. Most patients with TH show no clinical symptoms and the condition is only revealed by medical tests; a small minority of patients have been diagnosed only during medical tests and operations for swelling of the thyroid gland. Hashimoto’s thyroiditis is an autoimmune disease. Again, most patients show no clinical symptoms, while a small minority show a mass on the thyroid gland. As far as we know, no patient showing absence of a thyroid lobe, Hashimoto’s thyroiditis, and congenital absence of the ipsilateral parathyroid gland found during the operation has been reported previously.

## Case presentation

**Fig. 1:**
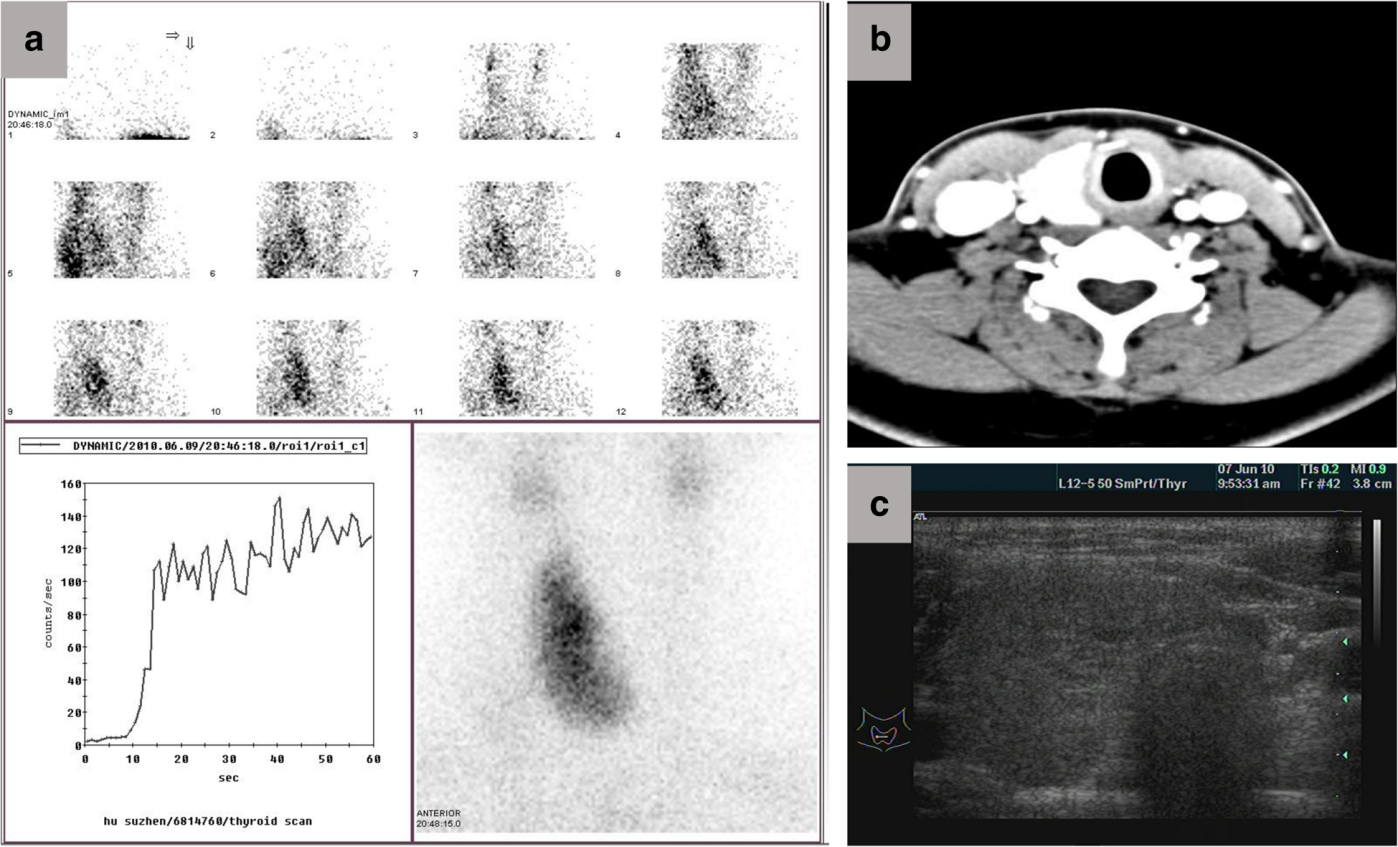
Augmentation of right lobe and absence of left lobe of thyroid **a**. Thyroid radionuclide imaging, **b**. CT scanning, **c**. Ultrasonic examination

**Fig. 2:**
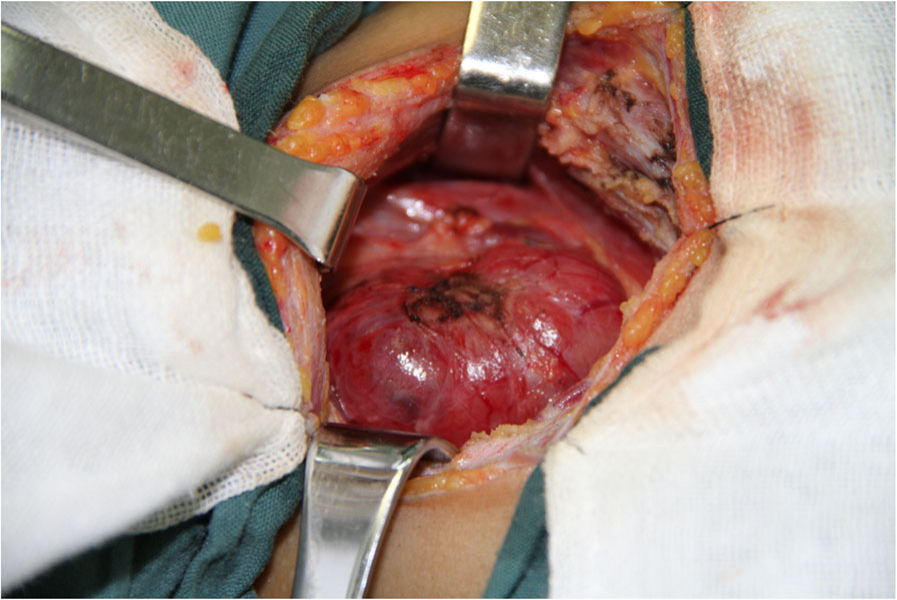
Swelling of right lobe and absence of left lobe found in the thyroid

**Fig. 3:**
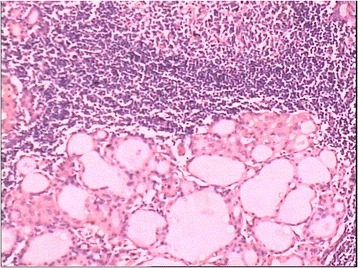
Atrophy of thyroid gland, eosinophilic change of epithelial cells and lymphocyte proliferation (HE ×200)

A 31-year-old woman was admitted to our hospital because of a mass on the neck. Examination revealed a 3-cm mass on the right neck with no pain and with movement during swallowing. Color Doppler Ultrasound revealed enlargement of the right lobe of the thyroid, tallish echo nodules, 19 × 11 mm, of uncertain nature, and the absence of images manifesting the left lobe ECT examination demonstrated the absence of images manifesting the left lobe and definite nodules on the right lobe in the middle of the thyroid. CT showed an enlarged right lobe and the absence of images manifesting the left lobe (Fig. 1) and showed no sign of tracheal compression. The antiTG and antiTPO levels were 45 and 8.5 IU/L, respectively, indicating normal thyroid and parathyroid gland function. The patient had no similar family history. In order to eliminate a tumor, the patient agreed to an operation but rejected needle biopsy. The operation revealed the absence of the left lobe of the thyroid and the left side of the parathyroid gland (Fig. 2). Diffuse enlargement and a hard nodule on the right lobe of the thyroid were found synchronously. The operation was immediately terminated when pathological examination of the thyroid nodule led to the diagnosis of Hashimoto’s thyroiditis (Fig. 3). The patient recovered well and accepted thyroxin tablets.

## Discussion

The thyroid gland is divided into two lobes that are linked by an isthmus located between them. In clinical practice, TH is very rare; its incidence is about 0.05–0.2% according to the medical literature [1, 2]. Of all patients showing absence of a thyroid lobe, 80% are female and 70% lack the left lobe [5]. During the course of diagnosis and treatment, patients with TH often show other thyroid diseases such as nodular goiter, hyperthyroidism, papillary carcinoma, etc. [6]. Increased risks of >thyroid pathology in TH patients include associated functional, morphological, and autoimmune thyroid disorders [7]. It is often difficult to diagnose TH combined with a mass on the thyroid, and the patients need surgery to complete the pathological diagnosis. Generally speaking, TH patients show accompanying absence of the ipsilateral parathyroid. Hence, a comprehensive examination must be completed before operating on patients with thyroid masses of unclear nature in order to avoid injury to the parathyroid gland. If the ipsilateral parathyroid gland were injured during excision of the ipsilateral lobe of the thyroid, with unclear absence of the other thyroid lobe, the patient would suffer lifetime disability by reason of persistent hypocalcemia resulting from permanent parathyroid dysfunction. Such cases combining TH with Hashimoto’s thyroiditis have rarely been reported in the literature. Although the cause of TH is unclear, it is generally considered to be related to a gene defect or the effect of environmental factors on a pregnant woman during embryo development. In some research, the PAX gene has been considered as a regulator of thyroid development, but no PAX gene mutations were found by Tonacchera et al., who examined the PAX gene in patients with congenital absence of a thyroid lobe [8].

Normally, the parathyroid gland has four lobes and is located dorsally between the true and false capsules of the bilateral lobus glandulae thyroideae; it is difficult to locate by imaging if it has normal function and volume. Hyperparathyroidism or parathyroidoma is topically diagnosed by obvious concentration of a radionuclide.

A patient with abnormal results on all preoperative examinations and revealing absence of the left lobes of the thyroid and parathyroid glands in the region located in the lobus glandulae thyroideae is considered to show absence of the ipsilateral parathyroid gland. However, the possibility of an ectopic parathyroid gland is not eliminated by imaging.

Hashimoto’s thyroiditis, an autoimmune disease also known as chronic lymphocytic thyroiditis, is accompanied by the presence of thyroid autoantibody in the blood plasma. The majority of patients with Hashimoto’s thyroiditis have no clinical symptoms, while about 20% show accompanying thyroid hypofunction and swelling of the thyroid gland, the main manifestation in symptomatic patients [9]. There are some reports in the literature that Hashimoto’s thyroiditis may be related to the occurrence of thyroid gland tumors. Patients with asymptomatic Hashimoto’s thyroiditis do not need treatment, but patients with thyroid hypofunction need hormone replacement therapy, though they generally do not need surgery. Only patients with larger masses that result in compression symptoms or suspicion of malignant need surgery. When the nature of the thyroid mass and the diagnosis are unclear, and the possibility of a tumor is not excluded by imaging, surgery is needed to confirm the diagnosis. Our patient accepted the operation and was diagnosed pathologically from tissue specimens because she refused needle biopsy or the biopsy gave no positive result, and the diagnosis was unclear. The patient lacking the left lobe of the thyroid accepted the operation despite Hashimoto’s thyroiditis because the nature of the phyma of the right thyroid lobe did not entirely eliminate the possibility of cancer.

It is often difficult to diagnose TH combined with a mass on the thyroid, and such patients need surgery in order to complete the pathological diagnosis. Patients lacking one lobe of the thyroid gland might show accompanying absence of the ipsilateral parathyroid gland [10]. So the patients with TH should accept systematic examination before the operation.

## Conclusions

Absence of thyroid lobe may accompany with a congenital absence of the ipsilateral parathyroid gland and Hashimoto’s thyroiditis. FNA is essential to diagnosis and decision-making of the treatment.

## Abbreviations

ECT: Emission Computed Tomography
TH: Thyroid hemiagenesis
